# Clinical characteristics and risk factors of liver injury in COVID-19: a retrospective cohort study from Wuhan, China

**DOI:** 10.1007/s12072-020-10075-5

**Published:** 2020-10-07

**Authors:** Ming Wang, Weiming Yan, Weipeng Qi, Di Wu, Lin Zhu, Weina Li, Xiaojing Wang, Ke Ma, Ming Ni, Dong Xu, Hongwu Wang, Guang Chen, Haijing Yu, Hongfang Ding, Mingyou Xing, Meifang Han, Xiaoping Luo, Tao Chen, Wei Guo, Dong Xi, Qin Ning

**Affiliations:** 1 Department and Institute of Infectious Disease, Tongji Hospital, Tongji Medical College, Huazhong University of Science and Technology, Wuhan, 430030 China; 2 Department of Paediatrics, Tongji Hospital, Tongji Medical College, Huazhong University of Science and Technology, Wuhan, China

**Keywords:** COVID-19, Liver damage, Clinical course, Alanine aminotransferase, Total bilirubin, Cytokine storm, Hypersensitive C-reactive protein, Neutrophil-to-lymphocyte ratio, Multivariate regression analysis, Retrospective cohort study

## Abstract

**Background:**

Coronavirus disease 2019 (COVID-19) has rapidly become a major international public health concern. This study was designed to evaluate the clinical characteristics and risk factors of COVID-19-associated liver injury.

**Methods:**

A fraction of 657 COVID-19 patients were retrospectively analyzed. Clinical and laboratory data were derived from electronic medical records and compared between patients with or without liver injury. Multivariate logistic regression method was used to analyze the risk factors for liver injury.

**Results:**

Among 657 patients, 303 (46.1%) patients had liver injury with higher rate in severe/critically ill patients [148/257 (57.6%)] than those in moderate cases [155/400 (38.8%)]. The incidence of liver injury was much higher in male [192/303 (63.4%)] than female [111/303 (36.6%)], and in severe/critical patients [148/303 (48.8%)] with percutaneous oxygen saturation ≤ 93% [89/279 (31.9%)] or peak body temperature ≥ 38.5 °C [185/301 (61.5%)] on admission. Liver injury-related inflammations included increased white blood cells, neutrophils and decreased lymphocytes. More patients with liver injury than without had increased serum IL-2R, TNFα, ferritin, hsCRP, PCT, ESR, γ-GT, and LDH. Multivariate regression analysis revealed that increasing odds of liver injury were related to male, higher serum hsCRP (≥ 10 mg/L), and neutrophil-to-lymphocyte ratio (NLR) (≥ 5). Moreover, more deceased patients (14/82 (17%)) had significantly elevated serum TBIL than discharged patients [25/532 (4.7%)].

**Conclusion:**

Liver injury is a common complication in COVID-19 patients. The potential risk factors of liver injury include male, hsCRP and NLR score. A close monitor of liver function should be warned in COVID-19 patients, especially in severe/critical individuals.

**Electronic supplementary material:**

The online version of this article (10.1007/s12072-020-10075-5) contains supplementary material, which is available to authorized users.

## Introduction

The ongoing outbreak of Coronavirus disease 2019 (COVID-19) has been recently becoming a pandemic [1]. At present, it is believed that SARS-CoV-2 mainly invades the respiratory system. Most patients have fever, cough, chest distress and dyspnea. Furthermore, systemic hyper-inflammation driving hypercoagulability is common in these patients [2].

However, accumulating evidences have suggested that some COVID-19 patients have different degrees of liver dysfunction [3–6]. Of 99 patients with COVID-19 enrolled in Wuhan Jinyintan Hospital, among whom 43 patients with different degrees of abnormal liver function were admitted to intensive care unit (ICU). One patient presented with obvious liver injury (alanine aminotransferase (ALT) 7590 U/L, aspartate aminotransferase (AST) 1445 U/L) [3]. Huang et al. showed that more patients admitted to ICU had elevated AST levels [4]. Wang et al. showed that patients admitted to ICU had significantly higher ALT levels [5].Moreover, Guan et al. extracted the currently largest cohort regarding 1099 mainly moderate SARS-CoV-2 infected patients and showed 39.4% with severe disease had elevated AST and 28.1% had elevated ALT, and the proportion was 18.2% and 19.8% in patients with non-severe disease [6].Given that the number of patients in these studies is relatively small, information about the clinical characteristics of liver injury in these patients is scarce. So far, to our knowledge, there are few reports that analyzed risk factors of liver injury in COVID-19 patients.

This current study aims to analyze the clinical characteristics of COVID-19 patients with liver injury, and evaluate the potential risk factors for the development of liver injury.

## Methods

### Study design and participants

All 657 patients included in the study were admitted from January 13, 2020 to February 25, 2020 in Tongji Hospital. The clinical characteristics and outcomes (death or discharge) were monitored up to March 28, 2020. Tongji Hospital was urgently reconstructed and has been assigned by Chinese government as a designated hospital for severe or critically ill COVID-19 patients. According to the guidance provided by the Chinese National Health Commission,all confirmed COVID-19 patients were diagnosed by SARS-CoV-2 nucleic acid detection of respiratory tract specimens.

### Data collection

The clinical data and laboratory data of patients were obtained from electronic medical records. The clinical data included sex, age, percutaneous oxygen saturation, heart rate, respiration rate, peak temperature during the course of the disease, duration of fever,comorbidities and treatment in the patients. The laboratory data included blood routine examination, liver function, renal function, coagulation function, creatine kinase (CK), creatine kinase-MB (CK-MB), hypersensitive cardiac troponin I (cTnI), N terminal pro-brain type natriuretic peptide (NT-proBNP), hypersensitive C-reactive protein (hsCRP), erythrocyte sedimentation rate (ESR), procalcitonin (PCT), ferritin, cytokines (interleukin 1β (IL-1β), interleukin 2 receptor (IL-2R), interleukin 6 (IL-6), interleukin 8 (IL-8), interleukin 10 (IL-10), tumor necrosis factor α (TNFα)),hepatitis B surface antigen and hepatitis C virus antibody examinations.

All patients were classified as being moderate, severe, or critically ill according to the Guidance for COVID-19 (6th edition) released by the National Health Commission of China [7] (Supp. Table S1). Liver injury was defined as serum level of ALT or total bilirubin (TBIL) greater than upper limit of normal value (ULN). The ULN of ALT or TBIL was 40U/L and 26 μmol/L, respectively, regardless of whether the patients had a history of chronic liver diseases.

### Statistical analysis

Continuous variables were described as median and interquartile range (IQR), and tested using non-parametric Mann–Whitney *U* test. Categorical variables were described as number and percentage, and tested using χ^2^ test, Fisher’s exact test, as appropriate. Multivariate logistic regression models were used to explore the risk factors for liver injury. Wilcoxon signed-rank test was used for paired continuous variables. SPSS (version 22.0) was used for all analyses. *p* value < 0.05 was considered statistically significant.

## Results

### Demographics and baseline characteristics of patients with or without liver injury

From January 13, 2020 to February 25, 2020, a fraction of 991 patients were enrolled from Tongji Hospital for this study. Among them, 657 patients with confirmed SARS-CoV-2 infection were subsequently included for further analyses. As of March 28, 2020, 532 patients recovered and were discharged, 82 patients died and 43 patients remained hospitalized.

As shown in Table 1, among 657 patients, 303 (46.1%) had liver injury. As shown in Table 2, the median age for all 657 patients was 63 years (IQR 49.0–70.0), of whom 347 patients (52.8%) were males. Male sex was more predominant in patients with liver injury [192/303 (63.4%)] than those without [155/354 (43.8%)]. 148 (48.8%) of liver injury patients were clinically diagnosed as severe or critically ill. Furthermore, percutaneous oxygen saturation ≤ 93% or peak body temperature ≥ 38.5 °C on admission was more predominant in patients with liver injury. Among those patients, the median time of duration of fever from onset of symptoms to hospital admission was 10.0 [ (IQR) 7.0–14.0] days, which tended to be longer than those without [9.0 (4.0–12.8) days]. Viral hepatitis was much more frequent among patients with liver injury [31/303 (10.2%)] than those without [21/354 (5.9%)]. And there was no difference in metabolic disorder between patients with or without liver injury.

**Table 1:** Liver injury in COVID-19 patients

	All patients (*n* = 657)	Moderate (*n* = 400)	Severe/critical (*n* = 257)	*p* value
Liver injury	303 (46.1)	155 (38.8)	148 (57.6)	< 0.001
*ALT, U/L*	31.0 (15.0–57.5)	25.0 (14.0–52.5)	41.0 (19.5–65.5)	< 0.001
≤ ULN	380 (57.8)	254 (63.5)	126 (49.0)	0.001
1–2ULN	180 (27.4)	99 (24.8)	81 (31.5)	
2–5ULN	83 (12.6)	43 (10.8)	40 (15.6)	
> 5ULN	14 (2.1)	4 (1.0)	10 (3.9)	
*TBIL, μ mol/L*	8.9 (6.6–12.4)	8.1 (6.2–11.0)	9.9 (7.6–15.3)	< 0.001
≤ ULN	625 (95.1)	389 (97.3)	236 (91.8)	0.003
1–2ULN	25 (3.8)	10 (2.5)	15 (5.8)	
> 2ULN	7 (1.1)	1 (0.3)	6 (2.3)	
*γ-GT, U/L*	38.0 (23.0–70.5)	35.0 (20.5–61.0)	45.0 (28.0–86.0)	0.001
≤ ULN	497 (75.6)	323 (80.8)	174 (67.7)	< 0.001
1–2ULN	104 (15.8)	53 (13.3)	51 (19.8)	
2–5ULN	48 (7.3)	21 (5.3)	27 (10.5)	
> 5ULN	8 (1.2)	3 (0.8)	5 (1.9)	

Data were expressed as median (IQR), or *n* (%). *p* values were calculated by Mann–Whitney *U* test, *χ*^2^ test, or fisher’s exact test, as appropriate. The ULN of ALT, TBil, γ-GT were 40 U/L, 26 μ mol/L and 71U/L, respectively

*ULN* upper limit of normal value

**Table 2:** Clinical characteristics and laboratory findings of COVID-19 patients on admission

	Normal range	All patients (*n* = 657)	Non-liver injury (*n* = 354)	Liver injury (*n* = 303)	*p* value
*Age, years*	–	63 (49.0–70.0)	64 (51.0–71.0)	62 (47.0–70.0)	0.200
≤ 50		177 (26.9)	87 (24.6)	90 (29.7)	0.327
50–65		182 (27.**7**)	100 (28.2)	82 (27.1)	
≥ 65		298 (45.4)	167 (47.2)	131 (43.2)	
*Sex*	–				< 0.001
Female		310 (47.2)	199 (56.2)	111 (36.6)	
Male		347 (52.8)	155 (43.8)	192 (63.4)	
*Clinical classification*	–				< 0.001
Moderate		400 (60.9)	245 (69.2)	155 (51.2)	
Severe/critical		257 (39.1)	109 (30.8)	148 (48.8)	
*Percutaneous oxygen saturation, %*	–				0.010
> 93		450/615 (73.2)	260/336 (77.4)	190/279 (68.1)	
≤ 93		165/615 (26.8)	76/336 (22.6)	89/279 (31.9)	
*Respiratory rate, breaths/minute*	–				0.084
< 30		589/649 (90.8)	324/350 (92.6)	265/299 (88.6)	
≥ 30		60/649 (9.2)	26/350 (7.4)	34/299 (11.4)	
*Heart rate, beat/minute*	–				0.177
≤ 100		487/653 (74.6)	270/352 (76.7)	217/301 (72.1)	
> 100		166/653 (25.4)	82/352 (23.3)	84/301 (27.9)	
*Peak body temperature,* *°C*	–				0.001
< 37.3		102/643 (15.9)	68/342 (19.9)	34/301 (11.3)	
37.3–38.5		194/643 (30.2)	112/342 (32.7)	82/301 (27.2)	
≥ 38.5		347/643 (54.0)	162/342 (47.4)	185/301 (61.5)	
Duration of fever, days		10.0 (5.0–13.0)	9.0 (4.0–12.8)	10.0 (7.0–14.0)	0.005
Digestive symptoms		316 (48.1)	163 (46.0)	153 (50.5)	0.255
White blood cell count, × 10^12^/L	3.5–9.5	5.55 (4.31–7.47)	5.20 (4.03–6.73)	6.07 (4.52–8.89)	<0.001
≥10		81/655 (12.4)	27 (7.6)	54/301 (17.9)	<0.001
Neutrophil count, × 10^9^/L	1.8–6.3	3.87 (2.73–5.87)	3.47 (2.50–4.88)	4.51 (3.00–7.12)	<0.001
Lymphocyte count, × 10^9^/L	1.1–3.2	0.94 (0.65–1.36)	1.03 (0.73–1.41)	0.84 (0.60–1.22)	<0.001
Monocyte count, × 10^9^/L	0.1–0.6	0.41 (0.30–0.57)	0.41 (0.31–0.57)	0.41 (0.28–0.58)	0.533
Platelet count, × 10^9^/L	125–350	212 (156–280)	217 (159–273)	205 (150–282)	0.247
Hemoglobin, g/L Aspartate aminotransferase> 40, U/L	130–175 ≤ 40	129.0 (117.0–139.0) 176/657 (26.8)	126 (114–135) 38/354 (10.7)	133 (121–142.8) 138/303 (45.5)	<0.001 <0.001
Albumin< 35, g/L	35–52	374/656 (57.0)	174/353 (49.3)	200/303 (66.0)	< 0.001
Lactic dehydrogenase,U/L	135–225	292.0 (228.0–422.0)	259.0 (208.5–334.0)	340.0 (272.0–463.0)	< 0.001
Creatinine, μmol/L	59–104	70.0 (56.0–87.0)	66.0 (55.0–84.0)	75.0 (59.0–89.0)	0.002
Creatine kinase, U/L	≤ 190	82.0 (50.0–149.0)	75.0 (43.5–119.5)	94.0 (54.0–194.0)	0.011
Creatine kinase-MB, U/L	≤ 7.2	0.70 (0.40–1.30)	0.6 (0.38–1.10)	0.9 (0.40–2.05)	0.005
γ-Glutamyl transpeptidase> 71, U/L	10–71	109/656 (16.6)	23/353 (6.5)	86/303 (28.4)	< 0.001
Alkaline phosphatase> 130, U/L	40–130	42/656 (6.4)	4/353 (1.1)	38/303 (12.5)	< 0.001
Prothrombin time,,seconds	11.5–14.5	14.0 (13.4–14.8)	13.9 (13.3–14.7)	14.1 (13.4–15.1)	0.013
Hypersensitive Troponin I, pg/ml	≤ 15.6	5.4 (2.3–13.9)	5.6 (2.30–12.6)	5.4 (2.4–16.1)	0.337
N terminal pro-brain type natriuretic peptide, pg/mL	< 116	162.0 (48.0–479.5)	142.0 (41.5–453.5)	194.0 (56.0–580.0)	0.057
Ferritin, μg/L> 400	30–400	187/253 (73.9)	78/125 (62.4)	109/128 (85.2)	< 0.001
Hypersensitive C-reactive protein ≥ 10, mg/L	< 10	450/608 (74.0)	220/334 (65.9)	230/274 (83.9)	< 0.001
Erythrocyte sedimentation rate, mm/h	0–15	37.0 (20.0–63.5)	35.0 (17.0–62.0)	38.0 (24.3–64.0)	0.038
Procalcitonin ≥ 0.5, ng/mL	0.02–0.05	40/514 (7.8)	12/253 (4.7)	28/261 (10.7)	0.011
Interleukin 1 β ≥ 5, pg/mL	< 5	70/444 (15.8)	39/233 (16.7)	31/211 (14.7)	0.555
Interleukin 2 receptor > 710, U/mL	223–710	200/443 (45.1)	87/233 (37.3)	113/210 (53.8)	0.001
Interleukin 6 ≥ 7, pg/ml	< 7	272/444 (61.3)	134/233 (57.5)	138/211 (65.4)	0.088
Interleukin 8 ≥ 62, pg/ml	< 62	40/443 (9.0)	20/233 (8.6)	20/210 (9.5)	0.730
Interleukin 10 ≥ 9.1, pg/mL	< 9.1	143/443 (32.3)	77/233 (33.0)	66/210 (31.4)	0.716
Tumor necrosis factor α ≥ 8.1, pg/ml	< 8.1	233/443 (52.6)	106/222 (47.7)	127/210 (60.5)	0.002
Metabolic disorder	–	304 (46.3)	165 (46.6)	139 (45.9)	0.850
Viral hepatitis	–	52 (7.9)	21 (5.9)	31 (10.2)	0.042
Hepatitis B		34 (5.2)	17 (4.8)	17 (5.6)	0.641
Hepatitis C		18 (2.7)	4 (1.1)	14 (4.6)	0.006

Data were expressed as median (Interquartile Range, IQR) or *n* (%). *p* values were calculated by Mann–Whitney *U* test, *χ*^2^ test, as appropriate. Patients with at least one of the following three conditions are classified as metabolic disorders: 1. Diabetes mellitus, 2. Hypertension, 3. Dyslipidemia

### Laboratory findings of patients with or without liver injury

As shown in Table 1, liver injury appeared in 277 (42.2%) of 657 patients with ALT elevation and in 32 patients (4.9%) with TBIL elevation, 160 (24.4%) of 657 patients had γ-GT elevation. There were significant differences in laboratory findings between COVID-19 patients with or without liver injury, including higher white blood cell, neutrophil and lower lymphocyte count in liver injury patients than those without. More [54/301 (17.9%)] patients with liver injury developed leukocytosis (white blood cell count ≥ 10 × 10^12^/L) than those [27/354 (7.6%)] without. There were no differences in NT-proBNP and cTnI levels between patients with or without liver injury (Table 2).

Elevated hsCRP (> 10 mg/L) appeared more frequent in patients with liver injury [230/274 (83.9%)] than those without [220/334 (65.9%)]. Moreover, serum ESR level was significantly higher in patients with liver injury compared with those without. In terms of cytokines, more patients with liver injury had elevated serum IL-2R, IL-6 and TNFα than those without.

To further investigate the correlation of inflammation with liver injury, a series of inflammation-based scores including mGPS, PLR, NLR, and CAR, LMR, PNI scores were calculated and compared (Table 3). More patients with liver injury have mGPS score over 2 points than those without. The PLR, NLR, and CAR scores were significantly higher in patients with liver injury than those without. The LMR and PNI scores were significantly lower in patients with liver injury than those without.

**Table 3:** Inflammation-based scores of COVID-19 patients on admission

	All patients (*n* = 657)	Non-liver injury (*n* = 354)	Liver injury (*n* = 303)	*p* value
*mGPS*				< 0.001
0	157/607 (25.9)	113/333 (33.9)	44/274 (16.1)	
1	140/607 (23.1)	79/333 (23.7)	61/274 (22.3)	
2	310/607 (51.1)	141/333 (42.3)	169/274 (61.7)	
PLR	212.3 (150.0–320.0)	205.2 (146.1–301.7)	226.9 (163.3–354.8)	0.004
NLR	3.82 (2.26–7.46)	3.30 (1.87–6.12)	5.30 (3.12–9.76)	< 0.001
LMR	2.38 (1.56–3.42)	2.54 (1.72–3.57)	2.15 (1.44–3.21)	0.001
PNI	36.55 (33.35–41.69)	37.85 (34.08–43.00)	35.50 (32.40–39.10)	< 0.001
CAR	1.08 (0.26–2.60)	0.68 (0.15–2.06)	1.52 (0.62–3.40)	<0.001

Data were expressed as median (IQR), or n (%). *p* values were calculated by Mann–Whitney *U* test, *χ*^2^ test, as appropriate

*mGPS* modified glasgow prognostic score, *PLR* platelet to lymphocyte ratio, *NLR* neutrophil to lymphocyte ratio, *LMR* lymphocyte to monocyte ratio, *PNI* Prognostic Nutritional Index, *CAR* C-Reactive to albumin ratio

The correlation between some surrogate markers of Systemic Inflammation Response Syndrome (SIRS), such as IL-2R, IL-6, TNFα, hsCRP, ferritin and PCT, and ALT, TBIL and γ-GT levels, was investigated. And the results showed serum ALT, TBIL and γ-GT all correlated with hsCRP and ferritin; meanwhile, TBIL and γ-GT correlated with IL-2R, TNFα and PCT (Supp. Table S2).

We also observed the effect of medications on liver injury. During hospitalization, some patients received one or more medications including antiviral drugs (Oseltamivir, Lopinavir/Ritonavir, or Arbidol), interferon inhalation, antibiotics, systemic glucocorticoid and non-steroidal anti-inflammatory drugs (NSAIDs). More patients [275/354 (77.7%)] in non-liver injury received Arbidol than that in liver injury patients [209/303 (69.0%)], whereas more patients with liver injury [176/303 (58.1%)] than those without [138/354 (39.0%)] received glucocorticoid therapy (Supp. Table S3).

### Liver injury in moderate and severe/critical patients

As shown in Table 1, more patients in severe/critical patients [148/257 (57.6%)] had liver injury than those in moderate ones [155/400 (38.8%)].The proportion of cases with ALT level above 1–2 or 2–5 times the ULN was higher in severe/critical patients than in moderate patients. In 3.9% (10/257) severe/critical patients, the ALT level was 5 times more than the ULN, and the highest ALT level reached 728 U/L. In comparison, only 1% (4/400) in moderate patients had an ALT level of 5 times more than the ULN and the highest was 385 U/L. The severe/critical patients with TBIL level of 1–2 or above 2 times the ULN accounted for 5.8% (15/257) or 2.3% (6/257), and the highest TBIL level was 174.1 μmol/L, whereas the moderate patients accounted for 2.5% (10/400) and 0.3% (1/400), and the highest level was 89.7 μmol/L.

The median γ-GT level was 35.0 (20.5–61.0) U/L in moderate patients and 45.0 (28.0–86.0) U/L in severe/critical patients. The proportion of cases with γ-GT level above 1–2, 2–5 or greater than 5 times the ULN was higher in severe/critical patients than those in moderate patients.

### Multivariate analysis for risk factors of COVID-19 associated liver injury

After considering the *p* value of univariate analysis, clinical significance and data integrity, 593 patients were enrolled and sex, metabolic disorder, viral hepatitis, hsCRP, NLR and peak temperature were selected as variables of logistic regression models. The independent risk factors for liver injury were as follows: male (OR 2.038, 95% CI 1.443–2.879, *p* < 0.001), hsCRP ≥ 10 mg/L (OR 1.733, 95% CI 1.118–2.687, *p* = 0.014), and NLR ≥ 5 (OR 2.154, 95% CI 1.486–3.124, *p* < 0.001) (Table 4).

**Table 4:** Multivariate analysis for risk factors in COVID-19 patients with liver injury

Variable	Univariable OR (95% CI)	*p* value	Multivariable OR (95% CI)	*p* value
Gender: Male vs. Female	2.221 (1.622–3.040)	< 0.001	2.038 (1.443–2.879)	< 0.001
Metabolic disorder	0.971 (0.714–1.321)	0.850	0.837 (0.589–1.188)	0.318
Viral hepatitis	1.807 (1.015–3.217)	0.044	1.615 (0.818–3.185)	0.167
Body temperate, °C: ≥ 38.5 vs.< 38.5	1.735 (1.267–2.377)	0.001	1.235 (0.975–1.565)	0.080
hsCRP, mg/L: ≥ 10 vs.< 10	2.709 (1.827–4.016)	< 0.001	1.733 (1.118–2.687)	0.014
NLR score: ≥ 5 vs.< 5	2.731 (1.977–3.773)	< 0.001	2.154 (1.486–3.124)	< 0.001

*p* values were calculated by logistic regression models

*OR* odds ratio, *CI* confidence interval

The dynamic changes of hsCRP and NLR with or without liver injury were further observed every 5 days after admission. Both hsCRP and NLR levels declined over the course of 15 days with higher values in patients with liver injury compared with those without (Supp. Figure S1).

### Dynamic changes of ALT and TBIL in deceased and discharged patients

**Fig. 1:**
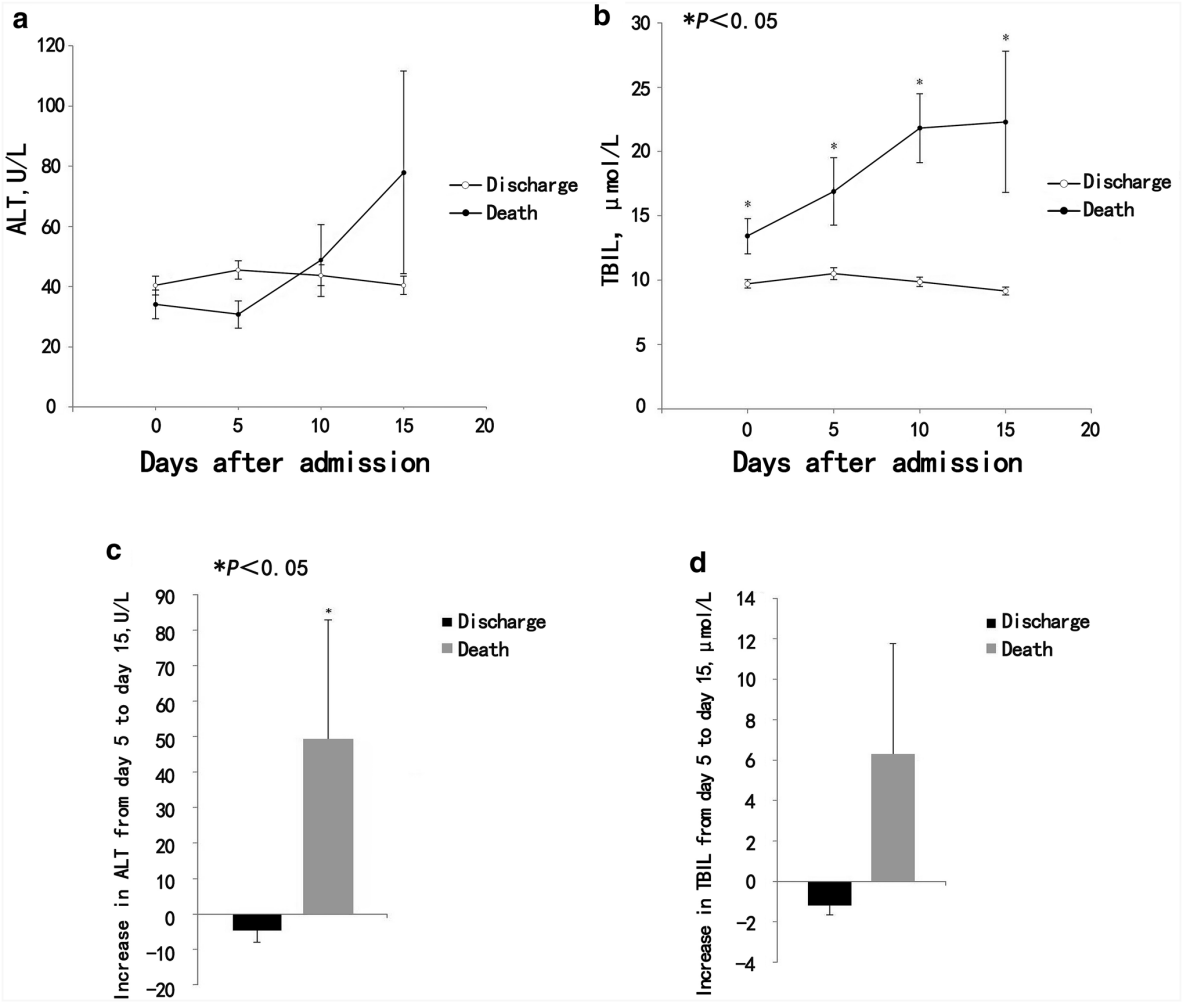
Dynamic profile of ALT and TBIL in deceased and discharged COVID-19 patients. **a** Dynamic changes in ALT; **b** Dynamic changes in TBIL. **c** Increase in ALT from day 5 to day 15 after admission. **d** Increase in TBIL from day 5 to day 15 after admission. *p* values were calculated by Mann–Whitney *U* test, or wilcoxon signed-rank test, as appropriate

To understand correlation of ALT and TBIL levels with disease progression, the dynamics of ALT and TBIL levels in 207 patients (21 deceased patients and 186 discharged patients) with complete data were analyzed from admission to the 15th day with an interval of every 5 days (Fig. 1a, b). During hospitalization, the ALT levels of the discharged patients slightly increased from admission to day 5, then gradually decreased thereafter until discharge. Whereas the ALT levels of those deceased patients rapidly increased from the 5th day after admission until day 15. TBIL levels of discharged patients stayed within normal range during the whole course, whereas it increased significantly in the deceased patients after admission, until day 15. The increase of ALT from day 5 to day 15 was significantly higher in deceased patients than those in discharged patients (Fig. 1c). Although the increases of TBIL levels in deceased patients from day 5 to day 15 were notable, there was no statistical significance compared with discharged patients (Fig. 1d).

Furthermore, TBIL levels of 1–2 or above 2 times the ULN [7/82 (8.5%) and 7/82 (8.5%)] in deceased patients were markedly higher than those in discharged patients [24/532 (4.5%) and 1/532 (0.2%)]. The ALT levels showed no significant differences between the two groups (Table 5).

**Table 5:** Analysis of ALT and TBIL in discharged or deceased COVID-19 patients

	Total (*n* = 614)	Discharged patients (*n* = 532)	Deceased patients (*n* = 82)	*p* value
*Peak ALT after onset*				0.742
Occurred within 7 days	19/258 (7.4)	16/221 (7.2)	3/37 (8.1)	
Occurred 7 days later	239/258 (92.6)	205/221 (92.8)	34/37 (91.9)	
*Peak TBIL after onset*				0.609
Occurred within 7 days	4/39 (10.3)	2/25 (8.0)	2/14 (14.3)	
Occurred 7 days later	35/39 (89.7)	23/25 (92.0)	12/14 (85.7)	
*ALT, U/L*				0.337
< ULN	356 (58.0)	311 (58.5)	45 (54.9)	
1–2ULN	167 (27.2)	141 (26.5)	26 (31.7)	
2–5ULN	79 (12.9)	71 (13.3)	8 (9.8)	
> 5ULN	12 (2.0)	9 (1.7)	3 (3.7)	
*TBIL, μ mol/L*				< 0.001
< ULN	575 (93.6)	507 (95.3)	68 (82.9)	
1–2ULN	31 (5.0)	24 (4.5)	7 (8.5)	
> 2ULN	8 (1.3)	1 (0.2)	7 (8.5)	

Data were expressed as *n* (%). *p* values were calculated by *χ*^2^ test, or fisher’s exact test, as appropriate. The ULN of ALT, TBIL were 40 U/L, 26 μ mol/L, respectively

*ULN* upper limit of normal value

## Discussion

The present study showed that liver injury was more prevalent in male, severe or critically ill patients with percutaneous oxygen saturation ≤ 93% or peak temperature ≥ 38.5 °C on admission, and comprehensively delineated the risk factors for COVID-19-associated liver injury. Liver injury-related inflammations included increased white blood cells, neutrophils and decreased lymphocytes. More patients with liver injury had increased serum IL-2R, TNFα, ferritin, hsCRP, PCT, ESR, γ-GT, and LDH. In addition, male, elevated hsCRP (≥ 10 mg/L) and NLR (≥ 5) were risk factors for COVID-19-associated liver injury. And patients with abnormal liver function tests (LFT) had higher risks of progressing to severe disease.

COVID-19-associated liver injury is defined as any liver damage occurring during disease progression and treatment of COVID-19 in patients with or without pre-existing liver diseases. In our cohort, there were 26 patients with elevated TBIL alone. According to data analysis of these patients, the results showed that the bilirubin elevation was due to direct bilirubin elevation as main body (15 cases) or both direct bilirubin and indirect bilirubin elevation (8 cases) or indirect bilirubin elevation as main body accompanied by the rise of AST (3 cases). Although all the 26 patients showed a degree of liver injury, Hemolytic Jaundice or Gilbert’s disease cannot be entirely excluded and requires further monitor in follow-up study.

Liver injury is not uncommon in previous studies in patients infected with severe acute respiratory syndrome coronavirus (SARS-Cov) and Middle East respiratory syndrome coronavirus (MERS-Cov) [8]. Likewise, abnormal LFTs were frequently seen in patients with COVID-19, with incidence rates exceeding 50% in some studies. Liver injury was presented in 30 out of 113 deceased patients from our previous reports [9]. However, patients with COVID-19 rarely develop acute or acute-on-chronic liver failure [4, 6, 9]. Taken together, these studies demonstrated that the majority of cases of COVID-19-associated liver injury occurred in severe or critically ill patients.

Consistent with the above findings, current study in a relatively larger cohort of severe/critical patients further demonstrated liver injury occurred more frequently in these patients [148/257 (57.6%)] than in moderate ones [155/400 (38.8%)]. Serum ALT, TBIL and γ-GT levels were markedly higher in severe and critically ill patients than in moderate patients, with a peak ALT of 728 U/L and peak TBIL of 174.1 μmol/L, respectively. The increase of ALT from day 5 to day 15 after admission was significantly greater in deceased patients than in discharged patients, and the proportion of cases in deceased patients with serum TBIL levels of 1–2 or above 2 times the ULN was markedly higher than that in discharged patients. These results provided the concrete evidence that impaired liver function was more frequent in severe and critically ill patients.

Moreover, multivariate regression analysis showed that hsCRP ≥ 10 mg/L, NLR ≥ 5 and male were the risk factors for liver injury in our study, which was consistent with Lei’s results [10]. As a traditional inflammatory marker, CRP is an acute-phase protein used for the diagnosis and follow-up of infection or tissue damage. Li et al. [11] showed CRP was independently associated with hepatic injury in patients with COVID-19. NLR, as a reliable marker of systemic inflammation and infection, has been considered as a predictor of bacterial infections, including pneumonia. Liu et al. [12] reported that NLR might predict severe illness in the early stage of COVID-19. Zhang et al. [13] identified NLR as an independent risk factor for severe COVID-19. Current studies indicated that there might be a sex predisposition to COVID-19, with men more prone to being affected likely due to much higher smoking rate in men [14].

Underlying mechanisms involved in liver injury in patients with COVID-19 are complex and interactive, including immune reconstitution in the presence of SARS-CoV-2, direct drug toxicity, systemic inflammation with liver involvement induced by cytokine storm or pneumonia-associated hypoxia [15]. Previous studies indicated that T lymphocytes particularly CD4^+^T and CD8^+^T cells decreased in severe COVID-19 patients [16], and immune function dysregulation in some cases may be associated with the development of acute respiratory distress syndrome (ARDS), collateral damage to multiple organs, such as liver, kidney and heart as well as death [17]. The exaggerated immune-mediated inflammation is known as “cytokine storm”. Similar to our study, accumulating evidence suggested that patients with severe COVID-19 might have a cytokine storm syndrome. Changes of inflammatory cytokines following SARS-CoV-2 infection may cause or contribute to liver damage. The underlying mechanism involved in cytokine accumulation in COVID-19 and subsequent liver injury warrants further investigation.

Recent study [18] demonstrated no significant increase in ALP and γ-GT in patients with COVID-19. In contrast, among 657 cases in the present study, more severely/critically ill patients [83/257 (32.3%)] than moderate patients [77/400 (19.3%)] had elevated γ-GT level. Previous pathological findings of COVID-19 in the liver showed moderate microvesicular steatosis and active inflammation in the hepatic portal area but no viral inclusions identified in the liver tissue [19]. The latest research showed that angiotensin-converting enzyme 2 (ACE2) was expressed in specific cholangiocytes in healthy liver tissues [20] and suggested liver injury in COVID-19 patients may not be only due to hepatocyte damage but cholangiocyte dysfunction. In our study, elevated γ-GT and bilirubin in patients with COVID-19 suggested that the virus may cause liver impairment by targeting cholangiocytes. The pathogenesis of COVID-19-associated liver injury warrants further investigation.

The impact of preexisting chronic liver diseases on the liver function of COVID-19 patients remains not fully elucidated. Ji et al. recently reported patients with non-alcoholic fatty liver disease (NAFLD) were at higher risk of disease progression, and developing abnormal liver function during hospitalization in COVID-19 patients [21]. Singh et al. [22] found COVID-19 patients with pre-existing liver disease, notably cirrhosis, were at higher risk for hospitalizations and mortality. Guan et al. reported that patients with preexisting chronic hepatitis B did not have more severe disease compared to the overall population [6]. Our study showed that 52 (7.9%) patients with underlying viral hepatitis (hepatitis B and hepatitis C)developed liver dysfunction, and more patients with underlying viral hepatitis developed liver injury than those without [31/303 (10.2%) vs 21/354 (5.9%)], which may be partly due to inflammatory responses and immune disorder of viral hepatitis. Further multivariate regression analysis showed that underlying viral hepatitis and metabolic disorder were not independent risk factors for liver injury, which is consistent with recent report [23], suggesting that liver injury cannot be entirely attributed to the preexisting chronic liver diseases in COVID-19 patients.

Considering the administration of antivirals, antibiotics, NSAIDS and glucocorticoids in the treatment of patients, potential drug-induced liver injury (DILI) deserves special concern. Given data provided is limited, based on recent results, we cannot draw a definitive conclusion about whether Lopinavir/Ritonavir increase the risk of developing liver damage [18, 24, 25]. Our study revealed a higher proportion of patients with liver injury had received systemic glucocorticoid. Nevertheless, we found a slightly reduced risk of hepatic injury in patients treated with Arbidol. This is likely due to the possibility that Arbidol may effectively suppress SARS-CoV-2 replication, which may be a beneficial pathway to lower the risk of the subsequent viral-induced liver injury. It has recently been reported that Arbidol combined with Lopinavir/Ritonavir achieved significantly higher rates of viral clearance and favorable clinical outcome compared with Lopinavir/Ritonavir used alone [26]. These findings further supported the hypothesis that effective antiviral therapy may prove useful in preventing or slowing the progression of the COVID-19 disease.

Our study has some limitations. First, nearly one third of critically ill patients developed disorders of consciousness on admission, which might result in a loss of some information (particularly a detailed history and subjective symptoms). Additionally, some physical examination and laboratory tests were not done in all the patients, and missing data or important tests might lead to bias of clinical characteristics. Second, the impact of COVID-19 in patients with preexisting chronic liver diseases, such as viral hepatitis, NAFLD, and alcohol-related liver disease, remains to be evaluated. Third, in the early period of epidemic, owing to some environmental constraints, such as the explosive growth of the infected population and the shortage of medical supplies, the results may be difficult to interpret accurately. More intensive surveillance or individually tailored therapeutic approach is needed for severe cases, especially among patients with liver or other important organ dysfunction.

In conclusion, immune-mediated inflammation may lead to COVID-19-associated liver injury. The potential risk factors for liver injury include male, hsCRP and NLR score. Moreover, patients with abnormal LFTs had higher risks of progressing to severe disease. Attention should be paid to monitor liver function during the course of COVID-19, especially in severely/critically ill patients. The data provided may be useful in helping us extend our understanding of the disease so that early effective intervention could be carried out promptly. Appropriate intervention and reasonable supportive care are expected to reduce the inflammatory response and then further reduce liver injury of patients with COVID-19.

## Electronic supplementary material

Below is the link to the electronic supplementary material.

Dynamic profile of CRP and NLR in COVID-19 patients with or without liver injury. A: Dynamic changes of hsCRP, B: Dynamic changes of NLR. *P* values were calculated by Mann-Whitney U test (JPEG 1070 kb)

Supplementary material 2 (DOCX 18 kb)
