# Supplementary figures and images for: Clinical characteristics and risk factors of liver injury in COVID-19: a retrospective cohort study from Wuhan, China
Source: Hepatol Int. 2020 Oct 7;14(5):723–32. doi: 10.1007/s12072-020-10075-5 (PMC7539280; doi:10.1007/s12072-020-10075-5)

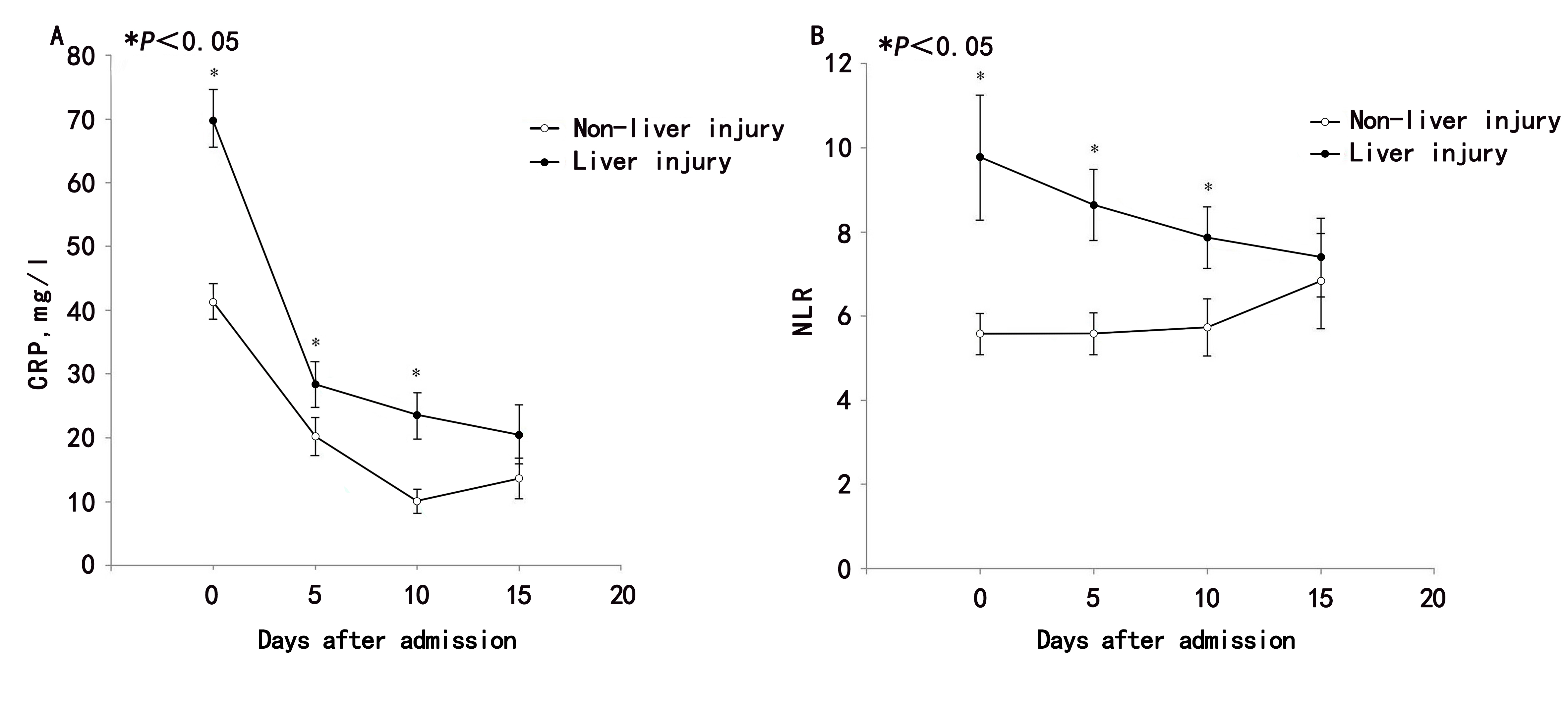

Supplement: Supplementary file 1 — Dynamic profile of CRP and NLR in COVID-19 patients with or without liver injury. A: Dynamic changes of hsCRP, B: Dynamic changes of NLR. P values were calculated by Mann-Whitney U test (JPEG 1070 kb) [file 12072_2020_10075_MOESM1_ESM.jpg]
