# Supplementary material for: Clinical characteristics and risk factors of liver injury in COVID-19: a retrospective cohort study from Wuhan, China
Source: Hepatol Int. 2020 Oct 7;14(5):723–32. doi: 10.1007/s12072-020-10075-5 (PMC7539280; doi:10.1007/s12072-020-10075-5)
Supplement: Supplementary file 2 — Supplementary material 2 (DOCX 18 kb) [file 12072_2020_10075_MOESM2_ESM.docx]

**Supplement**

**Methods**

**Data collection**

Modified glasgow prognostic score (mGPS) was constructed as following: patients with hsCRP level (>10 mg/l) and hypoalbuminemia (<35 g/l) were allocated a score of 2 points，with only hsCRP level (>10 mg/l) of 1 point, with normal hsCRP level (≤10 mg/l) of 0 point. Platelet to lymphocyte ratio (PLR) was calculated as platelet count divided by lymphocyte count, neutrophil to lymphocyte ratio (NLR) as neutrophil count divided by lymphocyte count, lymphocyte to monocyte ratio (LMR) as lymphocyte count divided by monocyte count. Prognostic nutritional index (PNI) =serum albumin (g/L) + 5 × lymphocyte count×10^9^/L. C‐Reactive to albumin ratio (CAR) was calculated as hsCRP divided by serum albumin.

| Moderate cases | Patients have symptoms like fever and respiratory tract symptoms, etc. and pneumonia manifestation can be seen in imaging. |
| --- | --- |
| Severe cases | Meeting any of the following:  1. Respiratory distress, respiratory rates ≥30 breaths/minutes;  2. The oxygen saturation ≤93% at a rest state;  3. Arterial oxygen tension (PaO2) over inspiratory oxygen fraction (FIO2) ratio ≤300 mm Hg;  4. Patients with >50% lesions progression within 24 to 48 hours in pulmonary imaging should be treated as severe cases. |
| Critical ill cases | Meeting any of the following:  1. Respiratory failure occurs and mechanical ventilation is required;  2. Shock occurs;  3. Complicated with other organ failure that requires monitoring and treatment in ICU. |

**Table S1. Clinical classification and definition.**

|  | ALT |  | TBIL |  | γ-GT |  |
| --- | --- | --- | --- | --- | --- | --- |
|  | r | *P* value | r | *P* value | r | *P* value |
| Hypersensitive C-reactive protein(mg/L) | 0.149 | <0.001 | 0.246 | <0.001 | 0.249 | <0.001 |
| Ferritin(μg/L) | 0.146 | 0.020 | 0.220 | <0.001 | 0.211 | 0.001 |
| Procalcitonin(ng/mL) | 0.006 | 0.895 | 0.178 | <0.001 | 0.091 | 0.040 |
| Interleukin 2 receptor(U/mL) | 0.011 | 0.814 | 0.229 | <0.001 | 0.161 | 0.001 |
| Interleukin 6( pg/mL) | 0.022 | 0.657 | 0.122 | 0.012 | 0.019 | 0.697 |
| Tumor necrosis factor α(pg/mL) | -0.004 | 0.940 | 0.257 | <0.001 | 0.249 | <0.001 |

**Table S2. The analysis of the correlation between some surrogate markers of SIRS(IL-2R, IL-6, TNFα, hsCRP, ferritin and PCT) and ALT, TBIL, and rGT levels**

|  | | | | |
| --- | --- | --- | --- | --- |
|  | **All patients**  **(n=657)** | **Non-liver injury**  **(n=354)** | **Liver injury**  **(n=303)** | ***P* value** |
| Oseltamivir | 137(20.9) | 71(20.1) | 66(21.8) | 0.587 |
| Lopinavir/Ritonavir | 161(24.5) | 79(22.3) | 82(27.1) | 0.159 |
| Arbidol | 484(73.7) | 275(77.7) | 209(69.0) | 0.012 |
| Interferon inhalation | 126(19.2) | 61 (17.2) | 65(21.5) | 0.171 |
| Antibiotics | 550 (83.7) | 287 (81.1) | 263 (86.8) | 0.046 |
| Systemic glucocorticoid | 314 (47.8) | 138 (39.0) | 176 (58.1) | <0.001 |
| NSAIDS | 96(14.6) | 45 (12.7) | 51(16.8) | 0.136 |

Data were n (%). *P* values were calculated by χ² test. NSAIDS: non-steroidal anti-inflammatory drugs.

**Table S3. Treatments of COVID-19 patients after admission.**
